# Succinylation Regulators Promote Clear Cell Renal Cell Carcinoma by Immune Regulation and RNA N6-Methyladenosine Methylation

**DOI:** 10.3389/fcell.2021.622198

**Published:** 2021-02-18

**Authors:** Wenqing Lu, Xiaofang Che, Xiujuan Qu, Chunlei Zheng, Xianghong Yang, Bowen Bao, Zhi Li, Duo Wang, Yue Jin, Yizhe Wang, Jiawen Xiao, Jianfei Qi, Yunpeng Liu

**Affiliations:** ^1^Department of Medical Oncology, The First Hospital of China Medical University, Shenyang, China; ^2^Key Laboratory of Anticancer Drugs and Biotherapy of Liaoning Province, The First Hospital of China Medical University, Shenyang, China; ^3^Liaoning Province Clinical Research Center for Cancer, Shenyang, China; ^4^Department of Pathology, Shengjing Hospital of China Medical University, Shenyang, China; ^5^Department of Respiratory and Infectious Disease of Geriatrics, The First Hospital of China Medical University, Shenyang, China; ^6^Department of Medical Oncology, Shenyang Fifth People Hospital, Shenyang, China; ^7^Marlene and Stewart Greenebaum Comprehensive Cancer Center, University of Maryland, Baltimore, MD, United States

**Keywords:** succinylation regulators, clear cell renal cell carcinoma, prognosis, immune, RNA N6-methyladenosine methylation

## Abstract

Succinylation is a newly discovered and multienzyme-regulated post-translational modification (PTM) that is associated with the initiation and progression of cancer. Currently, no systematic analyses on the role of succinylation regulators in tumors have been reported. In this study, we performed a comprehensive pan-cancer analysis on four well-known succinylation regulators (CPT1A, KAT2A, SIRT5, and SIRT7). We found that these regulators played specific and critical roles in the prognosis of clear cell renal cell carcinoma (ccRCC). We constructed a risk score (RS) based on two independent prognostic prediction factors, CPT1A and KAT2A, and subsequently developed a nomogram model containing the RS, which showed good accuracy in the prediction of overall survival (OS) in ccRCC patients. Furthermore, we used the similar expression pattern of four succinylation regulators according to consensus clustering analysis to divide the patients into three clusters that exhibited prominently different OS as well as clinicopathological characteristics. Differently expressed genes (DEGs) and pathway enrichment analyses of three clusters indicated that succinylation regulators might promote malignant progression of ccRCC by regulating the infiltration of immune cells and RNA N6-methyladenosine (m6A) methylation. Importantly, our data suggest that CPT1A and SIRT5 might up-regulate and down-regulate the expression of LRPPRC and EIF3B, respectively. Our study systematically analyzed the prognostic predictive values of four succinylation regulators and revealed their potential mechanisms in ccRCC aggressiveness. These data provide new insight into the understanding of succinylation modification and present clinical evidence for its role in ccRCC treatments.

## Introduction

Renal cell carcinoma (RCC) is one of the most common carcinomas with a continuously increasing incidence over several decades, in which clear cell renal cell carcinoma (ccRCC) accounts for approximately 75–80% (Shuch et al., 2015; Makhov et al., 2018). Curative resection is the most effective therapy for ccRCC. However, about 30% of patients could not be cured by surgical operation because of the local progression or distant metastasis at the first diagnosis, and around one third of patients suffered from recurrence after surgery (Li Q. K. et al., 2019). As ccRCC is not sensitive to radiotherapy or chemotherapy, the selection of appropriate therapeutic regimens for patients with advanced ccRCC remains challenging. The 5 years survival rate for patients with advanced ccRCC is only 11.7% (Siegel et al., 2017) and so there is an urgent need for the development of novel therapeutic options. Although new treatments such as anti-angiogenesis drugs and immune checkpoint inhibitors are recently recommended as first-line therapies, the objective response rate (ORR) is unsatisfactory yet (Angulo and Shapiro, 2019). It is necessary to develop novel prognostic biomarkers of ccRCC to screen out those patients with poor prognosis for more positive treatment.

Succinylation modification is a newly discovered post-translational modification (PTM) that regulates various physiological and pathological processes including tumor initiation and development. It is dynamically regulated by succinyl transferases including carnitine palmitoyltransferase 1A (CPT1A) (Kurmi et al., 2018) and lysine acetyltransferase 2A (KAT2A) (Wang Y. et al., 2017), and desuccinylases including Sirtuin5 (SIRT5) and Sirtuin7 (SIRT7). Accumulating evidence shows that succinylation regulators play important roles in tumor development by regulating the succinylation levels of substrate targets. CPT1A can promote the proliferation of breast cancer cells by succinylation of enolase 1 (Kurmi et al., 2018) and enhance metastasis of gastric cancer (GC) cells by succinylation of S100A10 (Wang et al., 2018). KAT2A has been shown to up-regulate 14-3-3ζ through its succinyltransferase activity which acts to promote proliferation, migration and invasion of human pancreatic ductal adenocarcinoma (PDAC) cells (Tong et al., 2020). SIRT5 can play a tumor-promoting role by desuccinylating substrates such as Cu/Zn superoxide dismutase (SOD1), pyruvate kinase M2 (PKM2), serine hydroxymethyltransferase2 (SHMT2), glutaminase (GLS), succinate dehydrogenase complex subunit A (SDHA) in the lung, liver, colon, breast and kidney cancers, respectively (Lin et al., 2013; Xiangyun et al., 2017; Chen et al., 2018; Greene et al., 2019; Ma et al., 2019). In particular, SIRT5 can promote breast cancer tumorigenesis in coordination with CTP1A (Kurmi et al., 2018; Greene et al., 2019). Also, SIRT5 can inhibit HCC tumorigenesis by regulating the activity of acyl-CoA oxidase 1 (ACOX1), or suppress GC invasion by desuccinylation of S100A10 (Wang et al., 2018). Based on these data, the roles of succinylation regulators in tumor development are complicated. Different succinylation regulators can have synergistic or antagonistic effects in specific tumors and the same succinylation regulator may have diverse functions in different types of tumors. There is a need to better understand the biology of succinylation regulators through a global analysis of their action in cancer development using the comprehensive bioinformatics approaches. However, such types of studies are not reported yet.

Succinylation regulators usually catalyze the succinylation modification of substrate proteins at lysine residues that are also frequently modified by other PTMs such as acetylation, ubiquitination and methylation. Compared to acetylation, succinylation can cause larger mass changes in substrate proteins due to the higher molecular weight of succinyl and can also have a greater effect on the charge of lysine residues from +1 to −1, resulting in more significant influences on the structure and function of target proteins (Kumar and Lombard, 2018). Moreover, competition between succinylation and other forms of PTMs at the same lysine residue can regulate the function of target proteins. For example, succinylation of S100A10 or GLS can increase the stability of these proteins by antagonizing ubiquitination and proteasome-dependent degradation (Wang et al., 2018; Zhao S. et al., 2019). The competitive relationship between succinylation and ubiquitination may regulate protein levels through the ubiquitin-proteasome pathway.

In this study, we conducted a pan-cancer analysis and identified a key role of succinylation regulators in ccRCC. Mechanistically, our results suggested that succinylation regulators might promote the malignant progression of ccRCC by regulating tumor immunity and m6A methylation regulators. This study offers a novel perspective on the role of succinylation regulators in ccRCC, provides useful insight into the screening of ccRCC patients for immune therapy and reveals a potential regulatory relationship between succinylation modification and RNA m6A methylation.

## Materials and Methods

### Study Design and Data Screening

All bioinformatics analyses in this study were performed following a flowchart as shown in Supplementary Figure S1. The RNA-seq transcriptome data and corresponding clinical information of pan-cancer including 33 tumors were acquired from the UCSC Xena Website^1^. The transcriptome data and more detailed clinical information of KIRC (also named ccRCC) were downloaded from The Cancer Genome Atlas (TCGA) database^2^ and normalized using the R program. A total of 251 samples with integral clinicopathological parameters, including T stage, N stage, M stage, survival status, overall survival (OS), age and gender, were divided into dataset 1 and a total of 242 patients with integral clinicopathological parameters except for N stage were contained in dataset 2. The baseline characteristics were highly similar in dataset 1 and dataset 2 except for gender, which was shown in Supplementary Materials (Supplementary Tables S1, S2 and Supplementary Figures S2A–F). The proteome data of ccRCC were obtained from The National Cancer Institute’s Clinical Proteomic Tumor Analysis Consortium (CPTAC) database^3^, in which 105 patients with expression information of CPT1A and KAT2A were screened to further analyze.

### Construction of Multi-Succinylation-Regulator Risk Score Model

Univariate and subsequent multivariate Cox regression analyses were performed to pick out the independent prognostic predictors among four well-known succinylation regulators by “survival” package. The risk score (RS) based on succinylation regulators for each patient was calculated with the expression values of the selected genes weighted by their corresponding coefficients according to the multivariate Cox regression analysis. Patients were then divided into high-risk and low-risk group by the median of RS and difference between two groups was analyzed by log-rank test and visualized by Kaplan-Meier survival curve.

### Establishment and Validation of Nomogram Prognostic Prediction Model

Multivariate Cox regression analysis was used to further pick out independent factors among RS and others clinical pathological characteristics, which were already screened by univariate Cox regression. According to the results of multivariate Cox regression analysis, the nomogram prognostic prediction model was established by the R package named “rms,” and the C-index of this model was calculated by “survival” package. Calibration curve was used to evaluate the performance of this model, and receiver operating characteristic (ROC) analysis was utilized to assess the accuracy of the model for survival prediction by “timeROC” package.

### Consensus Clustering on the Basis of Succinylation Regulators

To further investigate the function of succinylation regulators in ccRCC, patients in dataset 1 were separated into three clusters according to the best cut-off obtained from consensus clustering analysis using the R package named “ConsensusClusterPlus” (Wilkerson and Hayes, 2010). The in-group-proportion (IGP) statistic was used to evaluate the reproducibility of the consensus clustering by the “clusterRepro” package using data from dataset 2 (Kapp and Tibshirani, 2007; Li B. et al., 2019). Principal component analysis (PCA) within the R software was utilized to explore the expression patterns of genes in different clusters. Kaplan-Meier analysis was used to evaluate whether there was significant difference in OS among three clusters.

### Function Analysis Among Different Clusters

Differentially expressed genes (DEGs) in cluster 2 or cluster 1 compared to cluster 3 were screened by the “Limma” package (Ritchie et al., 2015) with the criteria of adjusted *P* < 0.05 and | Log fold change (FC)| > 0.585. Gene ontology (GO) and Kyoto Encyclopedia of Genes and Genomes (KEGG) pathway enrichment were performed by the package called “clusterprofiler” based on DEGs mentioned above (Yu et al., 2012).

### Immune Infiltration Analysis Among Different Clusters

The file of leukocyte gene signature matrix (LM22) and the corresponding source codes were downloaded from CIBERSORT website^4^ to assess the abundance of immune cells among clusters of ccRCC (Newman et al., 2015), the immune infiltration analysis was performed by R software (v3.6.3), only samples with *P* < 0.05 were retained for subsequent analysis.

### Construction of Immune Signature Underlining the Regulation of Succinylation

To explore the association of immune signature and succinylation regulators, a screening criteria was established as follows: (1) be up-regualted in cluster 2 as well as in clsuter1 in comparison with cluster 3; (2) be negatively correlated with OS of ccRCC patients (HR > 1, *P* < 0.05); (3) be contained in the gene list downloaded from Immport Shared Data website^5^. Then more powerful prognostic predictors from genes conformed to the criteria were filtered by Least Absolute Shrinkage and Selection Operator (LASSO) algorithm using “glmnet” package in R (Friedman et al., 2010) and finally constructed the immune signature associated with the succinylation regulators. RS_immune was reckoned with the expression values of the each gene contained in immune signature weighted by their coefficients according to LASSO Cox regression analysis.

### Succinylation Modification and Prognostic Value of m6A Regulators

The network of m6A regulators, which were reported by high-quality studies acquired from PubMed website^6^, was constructed using Cytoscape software (v3.6.1). Online website Protein Lysine Modifications Database (PLMD)^7^ was used to identify the succinylation and ubiquitination modified lysine residues within m6A regulators. In addition, the prediction value of m6A regulators in ccRCC was evaluated by univariate Cox regression analysis and LASSO.

### Validation by Immunohistochemistry From Clinical Specimens of ccRCC

CcRCC tissues and matched adjacent normal kidney tissues from 42 ccRCC patients received radical nephrectomy and confirmed by pathological diagnosis from June 2015 to June 2016 were collected in Shengjing Hospital of China Medical University. The baseline characteristics of 42 ccRCC patients for immunohistochemistry are shown in Supplementary Table S3. This study was approved by Ethics Committee of the First Hospital of China Medical University (AF-SOP-07-1.1-01). The expression pattern of CPT1A, KAT2A, SIRT5, SIRT7, LRPPRC, and EIF3B was assessed by immunohistochemistry using the paraffin embedded tissues. The intensity of staining was classified on a scale of 0–3: 0 (negative), 1, (weak), 2 (moderate), 3 (strong); and the heterogeneity of staining was scored as 0 (≤5%), 1 (6–25%), 2 (26–50%), 3 (51–75%), 4 (>75%). The protein expression of each molecule was calculated finally by the following formula: 3× scores of strongly staining cells + 2× scores of moderately staining cells + 1× scores of weakly staining cells. The expression pattern of each molecule was compared between normal and tumor tissues and then the correlation between CPT1A and LRPPRC, SIRT5 and EIF3B, CPT1A and EIF3B, SIRT5 and LRPPRC were calculated, respectively.

### Cell Culture

Human renal adenocarcinoma cell lines ACHN (TCHu199) was purchased from the Chinese Academy of Sciences (Shanghai, China) and cultured in RPMI-1640 medium with 10% fetal bovine serum (FBS) and 100 U/ml penicillin-streptomycin, under the optimum culture condition of 37°C and 5% CO_2_.

### Cell Transfection

The specific human siRNAs of CPT1A and SIRT5 were synthetized from JTS scientific (Wuhan, China). ACHN cells were inoculated into a six-well plate at the density of 1 × 10^5^ and siRNAs were transfected into cells by jetPRIME^®^ Transfection Reagent according to manufacturer’s instructions. The coding strands of negative control (NC) and different siRNAs are listed as follows:

NC siRNA: 5′-UUCUCCGAACGUGUCACGU-3′

siCPT1A-2: 5′-GGAUGGGUAUGGUCAAGAU-3′

siCPT1A-3: 5′-GCCUUUACGUGGUGUCUAA-3′

siSIRT5-1: 5′-GCAGAUUUUCGAAAGUUUU-3′

siSIRT5-3: 5′-GAGUCCAAUUUGUCCAGCU-3′

### Western Blotting

The protein samples were collected and quantified after transfected for 72 h. Samples were utilized to electrophoresis in an 8% SDS-polypropylene gel and then transferred onto PVDF membranes (Millipore, United States). After a 40 min-blocking with 5% skimmed milk, the membranes were incubated in primary antibodies for at least 6 h at room temperature and then washed by 1 × TBST buffer for 4 times. Finally, after incubation by secondary antibodies for 40 min and another washing for 4 times, the membranes were visualized using the Electrophoresis Gel Imaging Analysis System (DNR Bio-Imaging Systems, Israel).

### Antibodies

**Table:** 

Antibodies	Source	Identifier	Dilution Ratio	
			
			IHC	WB
CPT1A	Cell Signaling Technology	#12252		1:1,000
CPT1A	Proteintech	15184-1-AP	1:200	
KAT2A	Santa Cruz Biotechnology	sc-365321	1:50	
SIRT5	Sigma-Aldrich	HPA022002	1:800	1:500
SIRT7	Santa Cruz Biotechnology	12994-1-AP	1:50	
LRPPRC	Proteintech	21175-1-AP	1:400	1:1,000
EIF3β	Santa Cruz Biotechnology	sc-374156	1:50	1:2,000

### Statistical Analyses

All statistical analyses in the whole study except for immunohistochemistry were performed using R software (v3.6.3). Wilcoxon test was used to compare the expression level of genes between tumor and normal tissues, and one-way ANOVA test was utilized to compare the expression pattern of succinylation regulators and m6A regulators as well as the infiltration pattern of immune cells in patients with different clusters. Overall survival between different groups was analyzed by Kaplan-Meier survival curve with log-rank test. Correlation between genes expression was analyzed by Spearman correlation analysis. Relationship between protein expression and clinicopathological parameters was analyzed by chi-square test with package “stats” in R. Statistical analyses relating to immunohistochemistry was carried out by GraphPad Prism (v8.0.2), in which the unpaired Student’s *t*-test was used to analyze the expression pattern of each molecule between normal and tumor tissues, Pearson correlation was utilized to identify the relationship between CPT1A and LRPPRC, SIRT5 and EIF3B, CPT1A and EIF3B, SIRT5 and LRPPRC. *P* < 0.05 was defined statistically significant in the whole study.

## Results

### Altered Expression of Succinylation Regulators and Their Correlation With Clinicopathological Parameters of ccRCC Patients

**FIGURE 1:**
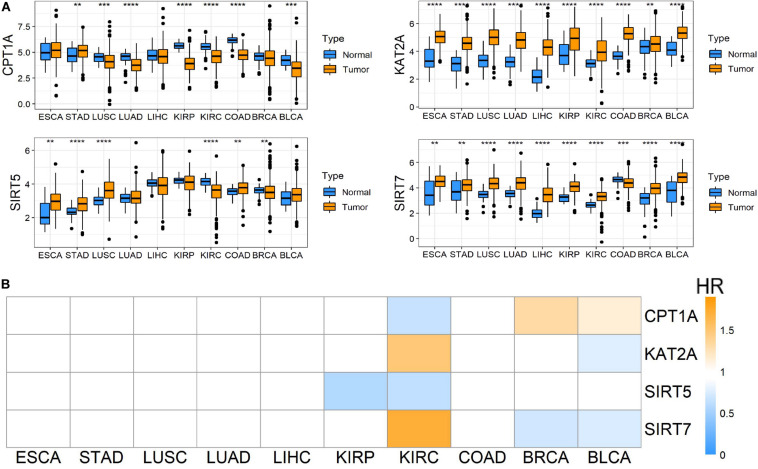
Pan-cancer analysis among 10 common tumors. **(A)** Different mRNA expression patterns of four succinylation regulators, CPT1A, KAT2A, SIRT5 and SIRT7, between tumor and normal tissues among 10 common tumors, *P < 0.05, **P < 0.01, ***P < 0.001, and ****P < 0.0001. **(B)** Heatmap of HR illustrating the association between each succinylation regulator and survival status in different tumors. Orange modules mean statistically significant HR > 1, blue modules mean statistically significant HR < 1, and white modules represent for the regulators with no significant prognostic prediction value. HR, hazard ratios; ESCA, esophageal carcinoma; STAD, stomach adenocarcinoma; LUSC, lung squamous cell carcinoma; LUAD, lung adenocarcinoma; LIHC, liver hepatocellular carcinoma; KIRP, kidney renal papillary cell carcinoma; KIRC, kidney renal clear cell carcinoma; COAD, colon adenocarcinoma; BRCA, breast invasive carcinoma; BLCA, bladder urothelial carcinoma.

To evaluate the roles of four well-known succinylation regulators (CPT1A, KAT2A, SIRT5, and SIRT7), we utilized the TCGA pan-cancer dataset to analyze the expression patterns and prognostic prediction values in 10 relatively common tumors including ESCA, STAD, LUSC, LUAD, LIHC, KIRP, KIRC, COAD, BRCA, and BLCA. We found that KAT2A was prominently up-regulated in all types of tumors compared to the corresponding normal tissues. SIRT7 was significantly up-regulated in 9 of 10 tumor types and down-regulated in COAD. The expression of CPT1A and SIRT5 showed significant differences between several tumors and normal tissues with a high level of heterogeneity in different tumors (Figure 1A). However, univariate Cox regression analysis showed that the overall survival (OS) of patients with KIRC (also named ccRCC), but not other tumor types, was associated with all four regulators (*P* < 0.05) (Figure 1B and Supplementary Table S4). Therefore, further analysis was focused on ccRCC. We performed correlation analysis between the expression of succinylation regulators and the clinicopathological parameters in dataset 1. Our results showed that expression of CPT1A or SIRT5 was negatively correlated with deeper tumor infiltration and distant metastasis, whereas the expression of SIRT7 exhibited opposite effect (Supplementary Table S5). The expression of KAT2A was not significantly correlated with any clinicopathological parameters. Taken together, these data indicated that succinylation regulators might play a more important role in the development and progression of ccRCC.

### Establishment of a Risk Score and Prognostic Predictive Nomogram Model Based on the Expression of Succinylation Regulators in ccRCC

**FIGURE 2:**
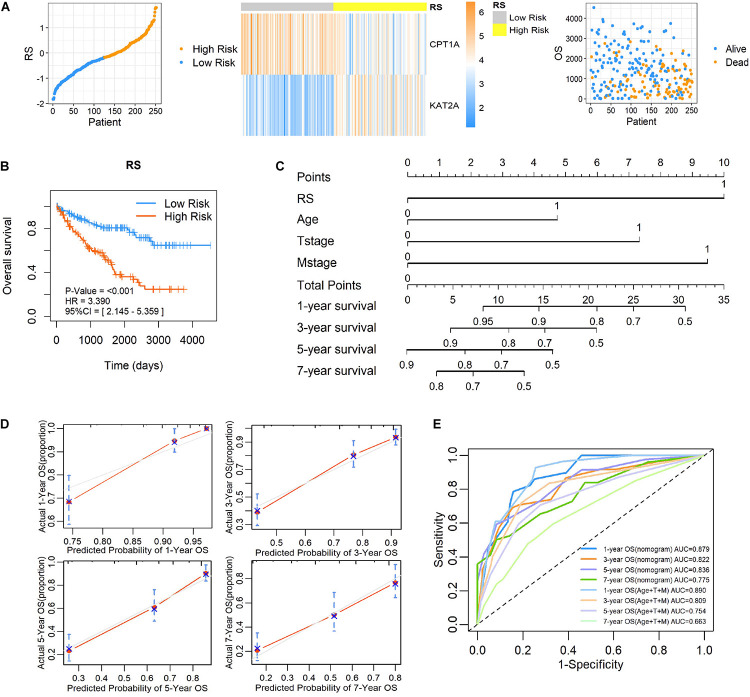
Establishment and validation of a risk score and nomogram prognostic prediction model based on succinylation regulators in dataset 1. **(A)** Distribution of the RS, OS, survival status, and the relative expression of CPT1A and KAT2A among ccRCC patients in dataset 1. **(B)** Kaplan-Meier survival curve for ccRCC patients with high and low RS.**(C)** The nomogram prognosis prediction model containing RS, Age, T stage and M stage. **(D)** The calibration plots suggested the comparison between predicted and actual outcome for 1-, 3-, 5-, and 7-year survival probabilities in the nomogram model. **(E)** ROC curves described the predictive ability of nomogram model and traditional model only containing age, T stage and M stage for 1-, 3-, 5-, and 7-year survival probabilities. RS, risk score; OS, Overall Survival; ROC, Receiver Operating Characteristic.

To further clarify the prognostic predictive value of succinylation regulators in ccRCC, we performed univariate and multivariate Cox regression analyses. The results indicated that CPT1A (HR = 0.535, 95%CI = 0.386–0.742, *P* < 0.001) and KAT2A (HR = 2.026, 95%CI = 1.404–2.922, *P* < 0.001) were the independent prognostic predictors for ccRCC (Table 1). Then, a risk score (RS) was calculated for each patient according to the following formula: RS = 0.706 × EXP[KAT2A] − 0.625 × EXP[CPT1A]. The patients were then divided into high- and low-RS groups based on the median of RS compared to the low-RS group, the high-RS group exhibited low expression of CPT1A, high KAT2A expression and reduced survival (Figure 2A). The Kaplan-Meier curve analysis showed that the OS of the high-RS group was notably shorter than the low-RS group (HR = 3.390, 95%CI = 2.145–5.359, *P* < 0.001) (Figure 2B). Similar results were found in dataset 2 (Supplementary Figures S3A,B). Furthermore, the relationship between RS and different clinical parameters was analyzed by a Chi-square test and showed that a high RS was positively associated with T stage (*P* = 0.012), M stage (*P* = 0.004), as well as male (*P* = 0.034, Table 2).

**TABLE 1:** The univariate and multivariate Cox regression analysis between four well-known succinylation regulators and OS in dataset 1.

	Univariate cox				Multivariate cox			
		
Gene	Coef	HR	95%CI	P	Coef	HR	95%CI	*P*
CPT1A	–0.584	0.558	0.421–0.739	<0.001	−0.625	0.535	0.386–0.742	<0.001
KAT2A	0.740	2.095	1.624–2.702	<0.001	0.706	2.026	1.404–2.922	<0.001
SIRT5	–0.601	0.548	0.307–0.979	0.042	−0.004	0.996	0.555–1.787	0.988
SIRT7	1.322	3.749	2.265–6.205	<0.001	0.016	1.016	0.487–2.116	0.967

**TABLE 2:** The correlation between RS and clinicopathological parameters in dataset 1.

Parameter	Total	Low RS	High RS	*P*
T stage				0.012
T1 + T2	148	84	64	
T3 + T4	103	41	62	
N stage				0.073
N0	235	121	114	
N1	16	4	12	
M stage				0.004
M0	209	113	96	
M1	42	12	30	
Age				0.849
<Median	123	60	63	
≥Median	128	65	63	
Gender				0.034
Female	99	58	41	
Male	152	67	85	

**The median age of ccRCC patients in dataset 1 is 62 years old.**

To estimate the survival probabilities of ccRCC patients at 1, 3, 5, and 7 years, we established a nomogram prognostic prediction model based on all independent prognostic predictors recognized by the univariate and multivariate Cox regression analyses including RS (HR = 3.082, 95%CI = 1.913–4.966, *P* < 0.001), T stage (HR = 2.278, 95%CI = 1.447–3.588, *P* < 0.001), M stage (HR = 2.876, 95%CI = 1.788–4.625, *P* < 0.001) and age (HR = 1.729, 95%CI = 1.138–2.626, *P* = 0.010, Table 3 and Figure 2C). The C-index of this model was 0.777, with a 95%CI ranging from 0.731 to 0.823. The overlapping of the calibration curve between the predictive values from the nomogram model and the actual observations demonstrated the accuracy of this model (Figure 2D). Next, we compared the performance of the nomogram model with the traditional model that only contained the clinical parameters (T stage, M stage and age) in the prognostic prediction. The results showed that the nomogram model was superior in predicting OS at 3, 5, and 7 years, whilst the traditional model had a larger area under the curve (AUC) for 1 year survival prediction (Figure 2E). These results suggested that the nomogram model was more powerful than the traditional model in predicting the long-term survival of ccRCC patients.

**TABLE 3:** The univariate and multivariate Cox regression analysis between RS and other clinicopathological parameters and OS in dataset 1.

	Univariate cox				Multivariate cox			
		
Parameter	Coef	HR	95%CI	*P*	Coef	HR	95%CI	*P*
T stage	1.148	3.151	2.075–4.784	<0.001	0.823	2.278	1.447–3.588	<0.001
N stage	1.233	3.430	1.818–6.472	<0.001	0.334	1.397	0.707–2.758	0.336
M stage	1.444	4.237	2.752–6.524	<0.001	1.056	2.876	1.788–4.625	<0.001
Age	0.450	1.568	1.035–2.376	0.034	0.547	1.729	1.138–2.626	0.010
Gender	0.021	1.021	0.674–1.547	0.921				
RS	1.221	3.390	2.145–5.359	<0.001	1.126	3.082	1.913–4.966	<0.001

**The median age of ccRCC patients in dataset 1 is 62 years old.**

### Cluster Identification Based on Consensus Clustering of Succinylation Regulators in ccRCC

**FIGURE 3:**
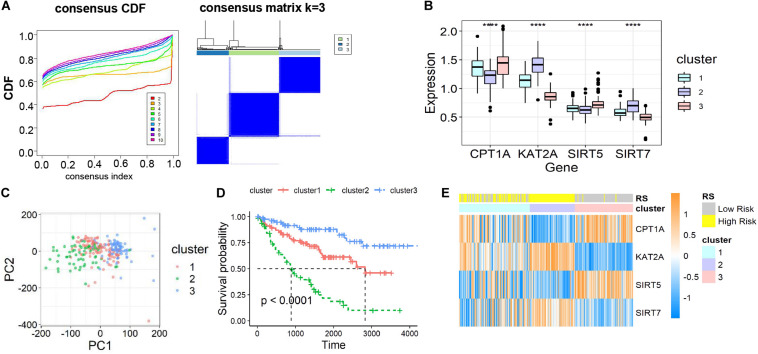
Identification and characteristic description of the consensus clusters based on succinylation regulators in dataset 1. **(A)** Consensus clustering CDF for k = 2 to 10 and consensus clustering matrix for k = 3. **(B)** The different expression pattern of four succinylation regulators in three clusters at mRNA level, ****P < 0.0001. **(C)** PCA of the total mRNA expression profile in dataset 1. **(D)** Kaplan-Meier survival curve for ccRCC patients in different clusters. **(E)** The heatmap demonstrated the expression of four succinylation regulators in patients contained in dataset 1 as well as the association between RS and consensus clusters. CDF, cumulative distribution function; PCA, principal component analysis.

To further ascertain the function of all succinylation regulators, consensus clustering analysis was performed to distinguish the expression similarities of four succinylation regulators. The patients in dataset 1 were separated into three clusters according to the minimum value of cumulative distribution function (CDF) (*k* = 3, Figure 3A). Significant differences in the expression patterns of the four regulators were found in clusters 1 (CPT1A^*medium*^, KAT2A^*medium*^, SIRT5^*medium*^, SIRT7^*medium*^), 2 (CPT1A^*low*^, KAT2A^*high*^, SIRT5^*low*^, SIRT7^*high*^), and 3 (CPT1A^*high*^, KAT2A^*low*^, SIRT5^*high*^, SIRT7^*low*^) (Figure 3B). Patients in each cluster gathered well in sub-classes partitioned by principal component analysis (PCA), verifying the rationality of the consensus clustering (Figure 3C). The patients in cluster 2 had the shortest OS amongst the three clusters, while patients in cluster 3 showed the longest OS (*P* < 0.001, Figure 3D). When comparing groups were divided by the RS and clusters, we found that cluster 2 was completely contained in the high-RS subgroup (Figure 3E). For the clinicopathological features, cluster 2 was positively related to later T stage (*P* < 0.001) and M stage (*P* = 0.001) (Table 4). Taken together, ccRCC patients could be successfully separated by consensus clustering and cluster 2 accurately identified patients with more malignant characteristics in the high-RS group.

**TABLE 4:** The correlation between clusters and clinicopathological parameters in dataset 1.

Parameter	Total	Cluster 1	Cluster 2	Cluster 3	*P*
T stage					<0.001
T1 + T2	148	63	24	61	
T3 + T4	103	39	41	23	
N stage					0.073
N0	235	98	57	80	
N1	16	4	8	4	
M stage					0.001
M0	209	87	45	77	
M1	42	15	20	7	
Age					0.854
<Median	123	48	32	43	
≥Median	128	54	33	41	
Gender					0.975
Female	99	41	25	33	
Male	152	61	40	51	
RS					<0.001
Low	125	45	0	80	
High	126	57	65	4	

**The median age of ccRCC patients in dataset 1 is 62 years old.**

The three-cluster classification was further verified using dataset 2. The in-group-proportion (IGP) values from cluster 1 to cluster 3 in dataset 2 were 0.972, 0.960 and 0.988, respectively (Supplementary Figure S4A). These values were much higher than a random partition into three clusters (IGP = 0.333). The three-clusters of dataset 2 highly resembled those of dataset 1 for the expression of four succinylation regulators (Supplementary Figure S4B), the distribution of patients in different principal components by PCA analysis (Supplementary Figure S4C), the relative length of OS time (Supplementary Figure S4D) and the relationship between cluster 2 and the high-RS group (Supplementary Figure S4E). In summary, ccRCC patients could be divided into three clusters according to the similarities in the expression of succinylation regulators. Poor prognosis was observed for clusters 1 or 2 and a good prognosis for cluster 3 was found.

### Association of Succinylation Regulators With Immune Cell Infiltration in ccRCC

**FIGURE 4:**
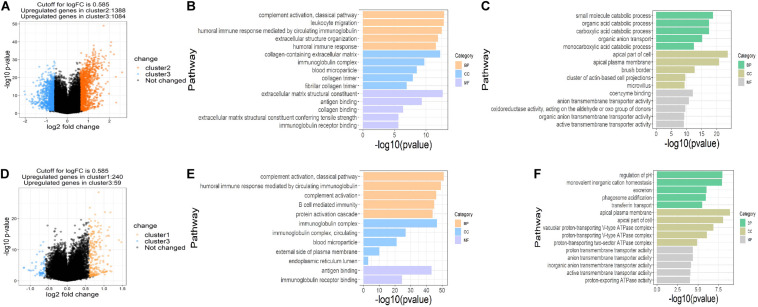
DEGs and enriched pathways in clusters 1 and 2 in comparison with cluster 3. **(A)** Volcano plot of DEGs in cluster 2 (*n* = 65) compared to cluster 3 (*n* = 84), the cutoff is 0.585 in the absolute value of logFC and 0.05 in *P-*value. **(B,C)** The top 15 up-regulated GO pathways **(B)** and top 15 down-regulated GO pathways in cluster 2 compared to cluster 3 **(C)**. **(D)** Volcano plot of DEGs in cluster 1 (*n* = 102) compared to cluster 3, the cutoff is 0.585 in the absolute value of logFC and 0.05 in *P-*value. **(E,F)** The top 12 up-regulated GO pathways **(E)** and top 15 down-regulated GO pathways in cluster 1 compared to cluster 3 **(F)**. DEGs, differently expressed genes; FC, fold change; GO, Gene Ontology.

To assess the functions of the three clusters, we screened the differently expressed genes (DEGs) between the clusters and performed the gene ontology (GO) pathway enrichment analysis. The DEGs between the clusters were screened with the restriction of an adjusted *P* < 0.05 and a | log fold change (logFC)| > 0.585. 1,388 up-regulated and 1,084 down-regulated genes in cluster 2, 240 up-regulated genes and 59 down-regulated genes in cluster 1 were identified relative to cluster 3, respectively (Figures 4A,D). GO pathway enrichment analysis of DEGs demonstrated the top 15 up-regulated and down-regulated pathways in clusters 1 and 2 (Figures 4B,C,E,F). The top 15 pathways were defined as the sum of the top five pathways in biological process (BP), cellular component (CC) and molecular function (MF), respectively, among which only two pathways in MF were significantly up-regulated in cluster 1. Interestingly, we found that most of the up-regulated pathways were related to immune regulation and part of the pathways overlapped between clusters 1 and 2, suggesting that succinylation regulators might promote the ccRCC by regulating immune pathways (Figures 4B,E).

**FIGURE 5:**
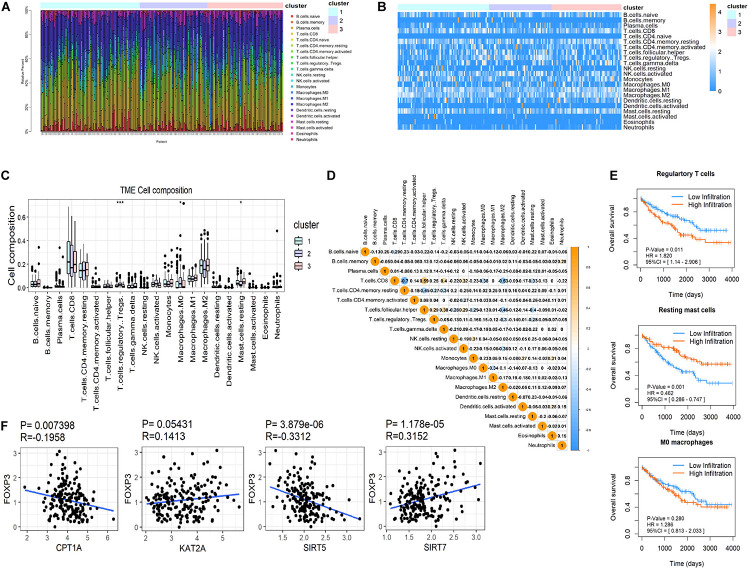
Immune infiltration in ccRCC among the three clusters. **(A,B)** Both the stacked column chart **(A)** and the heatmap **(B)** exhibited the proportion of 22 immune cells in 186 patients screened by CIBERSORT with a significant *P-*value (*P* < 0.05). **(C)** The infiltration pattern of immune cells (except for CD4 naive T cells) in different clusters, *P < 0.05 and ***P < 0.001. **(D)** Spearman correlation analysis of the 21 immune cells in 186 ccRCC patients. **(E)** Kaplan-Meier survival curves for patients with differently infiltrated Tregs, resting mast cells and M0 macrophages, respectively. **(F)** The Spearman correlation analysis between the expression of FOXP3 and the four succinylation regulators.

CIBERSORT was used to distinguish differences in immune infiltration among the three clusters. The result of 186 patients in dataset 1 met the requirements of CIBERSORT with a *P* < 0.05. The top 3 infiltrating immune cells were CD8^+^ T cells, M0 macrophages and resting memory CD4^+^ T cells, all of them showed no differences among the three clusters (Figures 5A–C). Interestingly, from a total of 22 immune cell types, three cell types were significantly different among the three clusters including regulatory T cells (Tregs, *P* < 0.001), M0 macrophages (*P* < 0.05) that exhibited an increasing trend with the promotion of malignancy, and resting mast cells (*P* < 0.05) that showed a decreasing tendency. The correlation analysis of 21 immune cell types (excluding naive CD4^+^ T cells that show no infiltration in any patient) revealed that CD8^+^ T cells were positively related to helper follicular T cells (*R* = 0.59, *P* < 0.05) and negatively related to resting memory CD4^+^ T cells (*R* = −0.7, *P* < 0.05, Figure 5D). Kaplan-Meier analysis indicated that infiltration of Tregs was associated with the poor prognosis of OS (HR = 1.820, 95%CI = 1.140–2.906, *P* = 0.011) and infiltration of resting mast cells was associated with longer OS (HR = 0.462, 95%CI = 0.286–0.747, *P* = 0.001). The infiltration of M0 macrophages had no predictive effect on the OS of ccRCC patients (Figure 5E). The infiltration of Tregs was significantly more different amongst the three clusters compared to resting mast cells. These results suggested that succinylation regulators might contribute to malignancy of ccRCC by enhancing the infiltration of Tregs and inducing an immunosuppressive microenvironment. To determine which succinylation regulators play a major role in the infiltration of Tregs, we performed a correlation analysis between each regulator and FOXP3, an essential gene marker for Tregs. The expression of FOXP3 was positively related to SIRT7 (*R* = 0.315, *P* = 0.007) and negatively related to SIRT5 (*R* = 0.331, *P* < 0.001) and CPT1A (*R* = −0.196, *P* < 0.001) (Figure 5F). These results suggested that SIRT7-high, SIRT5-low or CTP1A-low might contribute to the infiltration of Tregs.

### Establishment of a Succinylation Regulator-Related Prognostic Predictive Immune Signature in ccRCC

**FIGURE 6:**
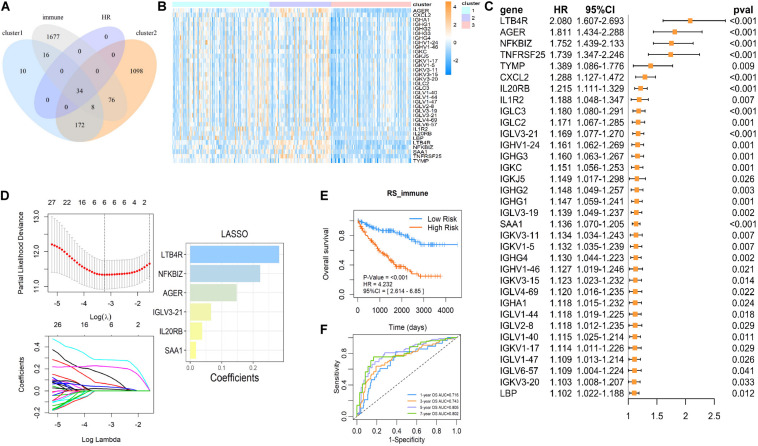
The immune signature based on succinylation modification. **(A)** The Venn diagram showed 34 candidates contained in up-regulated genes in cluster 2 relative to cluster 3, up-regulated genes in cluster 1 relative to cluster 3 and the immune gene list form Immport Shared Data website at the same time, and all have a significant HR > 1 (*P* < 0.05). **(B)** The heatmap displayed relative mRNA expression of 34 candidate immune genes in three clusters. **(C)** Univariate Cox regression analysis of the association between the mRNA expression of 34 candidate immune genes and OS of patients with ccRCC in dataset 1. **(D)** The coefficients of important prognosis prediction factors calculated by multivariate Cox regression using LASSO. **(E)** Kaplan-Meier survival curves for patients with high and low RS based on immune signature. **(F)** The ROC curves based on immune signature for 1-, 3-, 5-, and 7-year survival probabilities. LASSO, least absolute shrinkage and selection operator.

To further investigate the mechanism of succinylation regulators and immune regulation, we aimed to determine the key immune-related genes associated with succinylation regulators and ccRCC malignancy. Based on the criteria detailed in the “Materials and Methods” section, we identified a total of 34 immune-related genes that were up-regulated in clusters 1 and 2 and associated with poor prognosis (Figures 6A–C). Next, we performed the last absolute shrinkage and selection operator (LASSO) cox regression analysis to further identify six central immune factors as an immune signature (Figure 6D). Based on this immune signature, a RS was calculated according to the following formula: RS_immune = 0.148 × EXP[AGER] + 0.066 × EXP[IGLV3-21] + 0.038 × [IL20RB] + 0.283 × EXP[LTB4R] + 0.223 × EXP[NFKBIZ] + 0.018 × EXP[SAA1]. When separating the patients into the high- and low-risk groups based on the median of RS_immune, the OS of the two groups showed different trends (HR = 4.232, 95%CI = 2.614–6.850, *P* < 0.001) with the 5 and 7 years AUC larger than 0.8 (Figures 6E,F). Analysis of dataset 2 also showed similar results (Supplementary Figures S5A,B). All of these data indicated that succinylation regulators were associated with the expression of immune-related genes and a 6-gene immune signature could predict prognosis in ccRCC.

### Association of Succinylation Regulators With N6-Methyladenosine RNA Methylation in ccRCC

**FIGURE 7:**
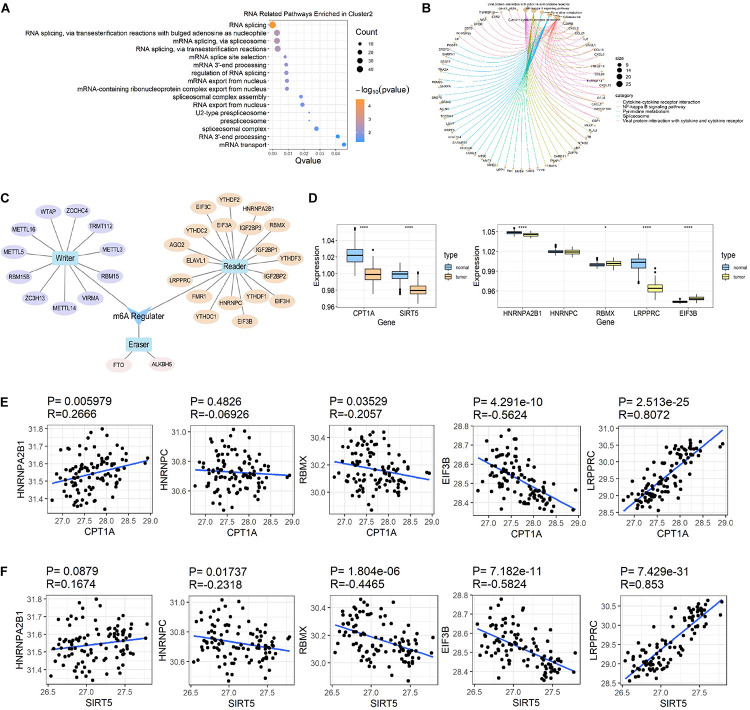
Succinylation regulators might impact the expression of some m6A regulators. **(A)** The bubble diagram displayed of all RNA related GO pathways enriched by up-regulated genes in cluster 2 compared to cluster 3. **(B)** The circle chart showed top 5 KEGG pathways enriched by up-regulated genes in cluster 2 compared to cluster 3. **(C)** A brief summary of 32 m6A regulators reported by high-quality articles. **(D)** The expression patterns of CPT1A, SIRT5, and 5 m6A regulators at protein level between ccRCC and normal tissues (Wilcoxon test), *P < 0.05, ****P < 0.0001. **(E)** The Spearman correlation analysis between the expression of CPT1A and 5 m6A regulators at protein level. **(F)** The Spearman correlation analysis between the protein expression of SIRT5 and 5 m6A regulators at protein level. GO, Gene Ontology; KEGG, Kyoto Encyclopedia of Genes and Genomes.

In addition to the immune-related pathways, abundant GO pathways relating to RNA modification were shown to be up-regulated (Figure 7A). The pathway named “spliceosome” was enriched in the top 5 of KEGG pathways (Figure 7B) in cluster 2 compared to cluster 3. These results suggested that RNA modification might also be associated with succinylation regulators and ccRCC malignancy. N6-methyladenosine (m6A) methylation, one of the most common RNA-related modifications, is catalyzed by m6A regulators (Zaccara et al., 2019). We hypothesized that succinylation regulators might regulate m6A regulators to affect RNA-related pathways. To determine whether m6A regulators could be succinylated, we searched the protein lysine modification database (PLMD) to seek succinylation modified sites in 32 m6A regulators including METTL3, METTL14, METTL16, WTAP, VIRMA, RBM15, RBM15B, ZC3H13, FTO, ALKBH5, YTHDF1, YTHDF2, YTHDF3, YTHDC1, YTHDC2, AGO2, RBMX, ELAVL1, HNRNPA2B1, HNRNPC, FMR1, LRPPRC, IGF2BP1, IGF2BP2, IGF2BP3, EIF3A, EIF3B, EIF3C, EIF3H, ZCCHC4, METTL5, and TRMT112 (Figure 7C). In total, 23 lysine sites from five m6A readers including HNRNPA2B1, HNPNPC, HNRNPG, LRPPRC, and EIF3B could potentially undergo succinylation modification (Table 5).

**TABLE 5:** The succinylation and ubiquitination modified lysine residues among 5 m6A regulators.

Protein	Succinylation modified lysine residue	Overlapped by ubiquitination	Peptides
HNRNPA2B1	120	Yes	KLFVGGIKDTEEHH
HNRNPC	176	Yes	GKSGFNSKGQRGSS
	197	No	GDDLQAIKELTQIK
	204	Yes	KKELTQIKKVDSLL
	223	Yes	KIEKEQSKAVEMKN
	243	Yes	EQSSSSVKDETNVK
	8	Yes	MASNVTNKDPRSMN
RBMX	217	Yes	RDDGYSTKSYSSRD
	30	Yes	ALEAVFGKGRIVEV
LRPPRC	107	Yes	IPKKLLQKFNDTCR
	1121	No	QVRRDYLKAVTTLK
	1224	No	GLAYLFRKIEEQLE
	1326	No	EAYNSLMKYVSEKD
	1332	No	MKSYVSEKVTSAKA
	1357	No	KLDDLFLKYASLLK
	187	Yes	SPTDFLAKEEANIQ
	613	Yes	QYFHQLEKNVKIPE
	649	Yes	AHLLVESKLDFQKT
	750	No	SAVLDTGKVGLVRV
	772	No	QDAINILKMKEKDV
	868	No	VLCKLVEKETDLIQ
	966	Yes	YNLLKLYKNGDWQR
EIF3B	729	Yes	KDLKKYSKFEQKDR

According to previous studies, succinylation regulators mainly regulate the activity or protein expression of their targeting molecules. We further analyzed the effects of succinylation regulators on the protein expression of the five m6A regulators using the ccRCC protein dataset from CPTAC. Based on their availability in the protein dataset, we selected CPT1A and SIRT5 as well as five m6A regulators (HNRNPA2B1, LRPPRC, RBMX, EIF3B, HNRNPC) for subsequent analyses. The protein expression of CPT1A and SIRT5 was lower in tumors compared to normal tissues (Figure 7D) and was highly similar to the mRNA expression patterns. The protein expression of the five m6A regulators was down-regulated or up-regulated in tumor tissues (Figure 7D). The CPT1A protein showed a positive correlation with HNRNPA2B1 (*R* = 0.267, *P* = 0.006) and LRPPRC (*R* = 0.807, *P* < 0.001), and was negatively correlated with RBMX (*R* = −0.206, *P* = 0.035) and EIF3B (*R* = −0.562, *P* < 0.001). CPT1A was not significantly correlated with HNRNPC (Figure 7E). The SIRT5 protein showed a negative correlation with HNRNPC (*R* = −0.232, *P* = 0.017), RBMX (*R* = −0.447, *P* < 0.001) and EIF3B (*R* = −0.582, *P* < 0.001), and a positive correlation with LRPPRC (*R* = 0.853, *P* < 0.001). SIRT5 was not significantly correlated with HNRNPA2B1 (Figure 7F). All the significant absolute *R*-values between CPT1A and LRPPRC, CPT1A and EIF3B, SIRT5 and EIF3B, SIRT5 and LRPPRC exceeded 0.5, suggesting that CPT1A and SIRT5 may regulate the expression of m6A regulators. From these data, we inferred that succinylation modification might promote malignant progression in ccRCC by regulating m6A regulators, at least partially by influencing protein expression levels.

### Importance of Succinylation Modified m6A Regulators in the Prognosis of ccRCC

**FIGURE 8:**
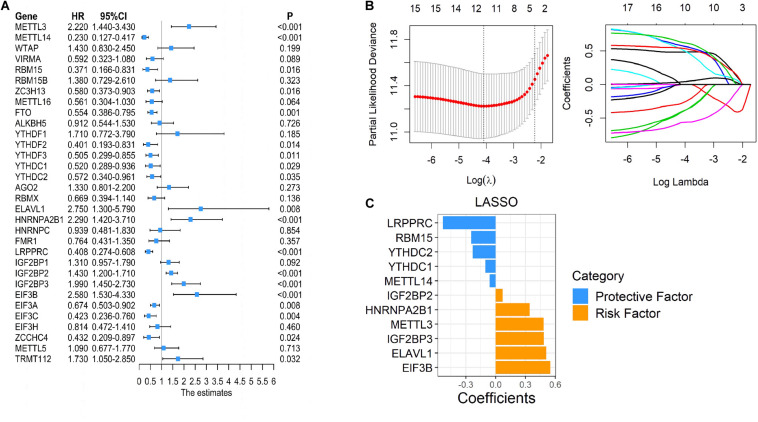
Succinylation-modified m6A regulators play important roles in the prognosis of ccRCC. **(A)** Univariate Cox regression analysis of 32 m6A regulators. **(B,C)** The detailed coefficients calculated by multivariate Cox regression using LASSO.

As many m6A regulators have been reported to be involved in the initiation and development of tumors, we aimed to test the prognostic predictive values of m6A regulators that may potentially be regulated by succinylation regulators in ccRCC. Global analysis using all 32 m6A regulators showed that 19 were significantly related to the OS of ccRCC patients (Figure 8A). To identify more powerful prognostic predictors amongst m6A regulators, we performed the LASSO Cox regression analysis and identified 11 genes (LRPPRC, RBM15, YTHDC2, YTHDC1, METTL4, TIF2BP2, HNRNPA2B1, METTL3, IGFBP3, ELAVL1, EIF3B) whose expression levels were associated with prognosis of ccRCC patients (Figures 8B,C). Importantly, three m6A regulators modified by succinylation including LRPPRC, EIF3B, and HNRNPA2B1, were present in the 11-gene list. LRPPRC and EIF3B were identified as the most important protective and risk factors with the largest absolute value of coefficients. These results suggested that m6A regulators, particularly those potentially regulated by succinylation regulators, have important prognostic values in ccRCC.

### Protein Level Validation of the Correlation Between Succinylation Regulators and Potential Downstream m6A Regulators

**FIGURE 9:**
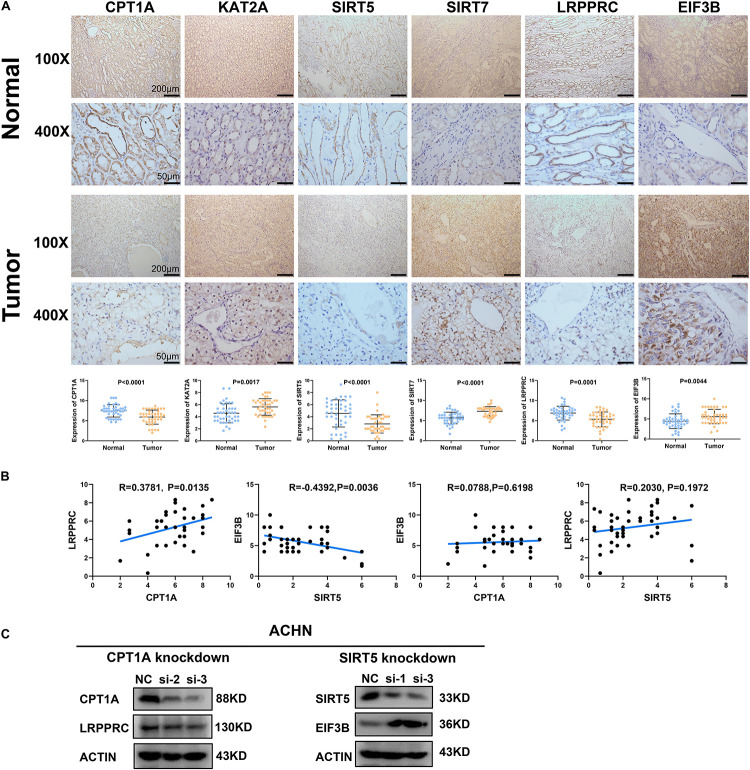
Validation of the correlation between succinylation regulators and m6A regulators at protein level. **(A)** The expression pattern of CPT1A, KAT2A, SIRT5, SIRT7, LRPPRC, and EIF3B between ccRCC tissues and corresponding normal tissues by immunohistochemistry. Error bars show standard error of the mean, and the middle bar represents the median expression level of each molecule. **(B)** The correlation analysis between CPT1A and LRPPRC, SIRT5 and EIF3B, CPT1A and EIF3B, SIRT5 and LRPPRC according to immunohistochemistry scores in 42 ccRCC tissues. **(C)** In ACHN cells, the expression of LRPPRC and EIF3B were detected by western blotting after transient knockdown of CPT1A and SIRT5, respectively.

To clarify the clinical relevance of our findings, we examined the expression of four succinylation regulators and their potential downstream m6A regulators in 42 pairs of ccRCC tissues and adjacent normal tissues by immunohistochemistry. The staining of CPT1A, SIRT5, and LRPPRC was significantly lower in ccRCC tissues compared to normal tissues, while the staining of KAT2A, SIRT7, and EIF3B was higher in ccRCC tissues than in the normal tissues (Figure 9A). These observations agreed with our previous analyses (Figures 1A, 7D). Importantly, a correlation analysis showed that the level of CPT1A was positively related to LRPPRC (*R* = 0.3781, *P* = 0.0135), whereas the level of SIRT5 was negatively related to EIF3B (*R* = −0.4392, *P* = 0.0036). No significant correlation was observed between CPT1A and EIF3B (*R* = 0.0788, *P* = 0.6198) or between SIRT5 and LRPPRC (*R* = 0.2030, *P* = 0.1972, Figure 9B). By combining the results from the CPTAC online dataset (Figures 7E,F) with our own data (Figure 9B), we deduced that CPT1A might increase the protein expression of LRPPRC whilst SIRT5 might inhibit the accumulation of EIF3B. Further validation was performed in the ACHN cell line by Western blotting, showing that LRPPRC was down-regulated following the silencing of CPT1A while EIF3B was prominently up-regulated by the knockdown of SIRT5 (Figure 9C). In sum, these results supported our bioinformatics analysis results that CPT1A might increase the expression of LRPPRC, while SIRT5 might decrease the expression of EIF3B in ccRCC.

## Discussion

In this research, we studied the prognostic predictive value of four succinylation regulators in ccRCC and explored the potential mechanisms of succinylation regulators in the progression of ccRCC. Until now, several studies have reported that CPT1A, SIRT5 and SIRT7 are individually related to the prognosis of ccRCC (Zhao Z. et al., 2019; Tan et al., 2020). Here, we took a comprehensive global analysis of the four succinylation regulators. According to our analyses, CPT1A and SIRT5 served as protective factors whereas SIRT7 acted as a risk factor for ccRCC, and these results were in agreement with previous studies (Zhao Z. et al., 2019; Tan et al., 2020). Also, we found that KAT2A could function as an independent risk factor for the prognosis of ccRCC. Importantly, the nomogram model involving both clinicopathological parameters and RS was superior in predicting long-term OS compared to a traditional model that only contains clinicopathological parameters. Therefore, succinylation regulators, in particular CPT1A and KAT2A, might serve as potential biomarkers for ccRCC due to their superior performance in OS estimation. Direct repression of CPT1A by HIF1 and HIF2 has been reported to reduce the transport of fatty acids into the mitochondria and force fatty acids to form lipid droplets for storage that can promote ccRCC (Du et al., 2017; Tan and Welford, 2020). The data reported in this study are consistent with previous reports, showing that CTP1A expression was down-regulated to promote ccRCC. SIRT5 has been reported to desuccinylate SDHA to promote ccRCC tumorigenesis (Ma et al., 2019). The findings in our study are inconsistent with this previous report as we found that SIRT5 was down-regulated in ccRCC. Nevertheless, succinylation modification is a multienzyme-regulated process in which the substrate protein might be simultaneously modified by several enzymes. Therefore, the investigation of a single succinylation regulator in previous studies may be insufficient to accurately reveal the role of succinylation modification in the development of ccRCC. To better understand the functions of succinylation modification in ccRCC, we analyzed all four succinylation regulators simultaneously to reveal their functions in ccRCC. We used the expression patterns of these regulators for consensus clustering analysis and divided ccRCC patients into three clusters. Similar to the nomogram prognostic model, the three clusters showed prominently different prognosis and clinicopathological characteristics. The patients in cluster 2 were completely included in the patients of the RS-high group, confirming the validity of the consensus clustering analysis. In contrast to the nomogram prognostic model using only CPT1A and KAT2A, the consensus clustering analysis considered the holistic effects of all the regulators and could more precisely reflect their functions in ccRCC. Furthermore, our pathway enrichment analyses of the three clusters suggested that succinylation regulators might play a role in the immune cell infiltration and m6A methylation in ccRCC.

We ascertained the prognostic value and the potential regulatory mechanism of succinylation regulators in ccRCC using a nomogram model and consensus clustering analysis. However, although this study suggested the comprehensive analysis of all the four regulators could help to find out some interesting discovery related to succinylation modification, bioinformatic analysis itself without experimental verification is hard to confirm if the prognostic prediction values of these molecules only depend on their succinylation regulatory activity. It is known that succinylation modification is not the only role of the four succinylation regulators. CPT1A is known to be able to regulate the oxidation of fatty acids (Tan and Welford, 2020); KAT2A is considered as classical acetylase both in histone and non-histone proteins, and its role of histone glutaryltransferase has also been discovered recently (Bao et al., 2019; Mutlu and Puigserver, 2020); SIRT5 is confirmed to own strong activity in demalonylase and deglutarylase while weak activity in deacetylase (Yang et al., 2017); SIRT7 acts as an deacetylase asl well as an “eraser” of the glutarylation of histone H4 lysine 91 (Bao et al., 2019). Therefore, more detailed investigations of these complex functions are required in future studies.

Our analyses suggested that the increased infiltration of Tregs in clusters 1 and 2 was associated with relatively short OS. The infiltration of Tregs was associated with poor prognosis and weak treatment response in ccRCC providing strong support for our results (Wang Y. et al., 2019; Pan et al., 2020). Moreover, our analyses implied that infiltration of Tregs in ccRCC might be associated with two desuccinylases, SIRT5 and SIRT7. Our results showed the positive relation of SIRT7 and negative relation of SIRT5 to FOXP3, which is an important marker of Tregs and critical for the immunosuppression function of Tregs. However, it remains unclear how SIRT5 and SIRT7 regulate the infiltration of Tregs. Loss of SIRT5 promotes the transcription of IL-1β in macrophages by increasing the succinylation level of PKM2 and forcing it to be translocated into the nucleus for the formation of the PKM2-HIF1α complex on the IL-1β promoter (Wang F. et al., 2017). As succinylation regulators do not directly regulate mRNA expression, SIRT5 might affect the infiltration of Tregs through immediate molecules such as the PKM2-IL-1β axis in ccRCC. As the accelerated proliferation of Tregs during immuno-therapy is known to reduce the efficacy of anti-PD-1 treatment (Kamada et al., 2019), SIRT5 and SIRT7 might have the potential to predict resistance to anti-PD-1 therapy. Also, the SIRT5 activator or SIRT7 inhibitor might have the potential to improve the efficacy of anti-PD-L1 therapy.

We identified a 6-immune gene signature that included LTB4R, NFKB2, AGER, IGLV3-21, IL20RB, and SAA1. These genes were overexpressed in clusters 1 and 2, and displayed excellent prognosis value in ccRCC. Among the six genes, SAA1 has been reported as a risk factor in ccRCC that can promote the proliferation of Tregs by inducing the secretion of IL-1β and IL-6 from monocyte (Nguyen et al., 2014; Wang Y. et al., 2019). Also, AGER and IL20RB have been reported as tumor promoters in ccRCC (Liu et al., 2020; Shen et al., 2020). LTB4R, NFKBIZ, and IGLV3-21 have not yet been explored in ccRCC. Our study revealed the excellent prognostic value of the 6-immune gene signature, highlighting the key role of succinylation regulators and immune regulation in ccRCC malignancy.

We found that RNA m6A methylation is another major pathway associated with succinylation regulators in ccRCC. RNA m6A methylation is the most widespread regulatory mechanism of RNA modification (Wang S. et al., 2017) and is dynamically regulated by three types of regulators containing “writers,” “erasers,” and “readers.” Writers and erasers contribute to the alteration of m6A methylation levels and readers control the regulatory effects of m6A modification on substrate molecules. RNA m6A methylation plays an important role in cancer development by regulating RNA splicing, stability, mRNA translational efficiency, secondary RNA structure, nuclear export and localization (Zaccara et al., 2019). Our results suggested that enrichment of RNA-related pathways in cluster 2 might be attributed to the regulation of m6A regulators by succinylation regulators. We identified 23 succinylation modified lysine residues among five m6A regulators including HNRHPA2B1, HNRNPC, RBMX, EIF3B, and LRPPRC. Interestingly, the five regulators all belonged to m6A “readers” in which HNRHPA2B1, HNRNPC, and RBMX mainly regulated the process of RNA splicing. These results were consistent with our findings that RNA splicing and spliceosome ranked as top pathways related to RNA in the GO and KEGG pathway enrichment (Figures 7A,B). By correlation analysis using the online ccRCC protein dataset (Figures 7E,F), analysis of our tissue specimens (Figure 9B) and preliminary validation in the ACHN cell line (Figure 9C), we confirmed that CPT1A and SIRT5 could up-regulate and down-regulate the expression of LRPPRC and EIF3B in ccRCC, respectively. We found that 14 of the 23 succinylation-modified lysine residues were also modified by ubiquitination containing five residues in LRPPRC and one residue in EIF3B (Table 5). In previous studies, a site-competition mechanism between succinylation and ubiquitination has been reported. For example, succinylation modification of GLS could inhibit ubiquitination via competition for same lysine residues leading to decrescent protein degradation and increscent protein stability (Zhao S. et al., 2019). These data suggest that high succinylation levels might contribute to the stability of proteins. According to this mechanism, we speculated that CPT1A might increase the stability of LRPPRC, while SIRT5 might reduce the stability of EIF3B in a succinylation-ubiquitination competition mechanism. However, these predictions require extensive validation in the laboratory. Our global analysis of 32 m6A regulators is more integral than any other previously published report and showed that succinylation-modified m6A regulators, especially LRPPRC and EIF3B, are important for the prognosis of ccRCC. Our online website and bioinformatic analyses showed the potential connection between succinylation modification and m6A methylation, providing novel insight into the relationships between the two epigenetic modifications. These bioinformatic analyses offer strong clues for future experimental validation.

## Conclusion

In conclusion, we discovered the prognostic value of succinylation regulators in ccRCC and established a nomogram prediction model, which showed good accuracy. Furthermore, the potential mechanism of succinylation regulators in ccRCC progression was revealed by regulating immune infiltration and RNA m6A methylation (Supplementary Figure S6). This study also provides clinical evidence for treatment options.

## Data Availability Statement

Publicly available datasets were analyzed in this study. This data can be found here: http://xena.ucsc.edu/, http://cancergemome.nih.gov/, https://proteomics.cancer.gov/programs/cptac.

## Ethics Statement

The studies involving human participants were reviewed and approved by Ethics Committee of the First Hospital of China Medical University. The patients/participants provided their written informed consent to participate in this study.

## Author Contributions

WL and XC designed the study and performed most of the bioinformatic analyses with the help of YL, JQ, and XQ. CZ, XY, and DW performed the immunohistochemistry and accomplished corresponding analysis. BB and ZL downloaded the datasets and offered suggestions for statistical analysis. YJ, YW, and JX helped to prepare figures and tables. WL wrote the manuscript under the guidance from YL, XC, and JQ. All authors revised, read, and approved the final manuscript.

## Conflict of Interest

The authors declare that the research was conducted in the absence of any commercial or financial relationships that could be construed as a potential conflict of interest.

## Footnotes

1 http://xena.ucsc.edu/

2 https://cancergenome.nih.gov/

3 https://proteomics.cancer.gov/programs/cptac

4 http://cibersort.stanford.edu

5 https://www.immport.org/shared/home

6 https://pubmed.ncbi.nlm.nih.gov/

7 http://plmd.biocuckoo.org/

## Abbreviations

ccRCC: clear cell renal cell carcinoma
m6A: N6-methyladenosine
PTM: post-translational modification
RS: risk score
OS: overall survival
DEGs: differently expressed genes
RCC: renal cell carcinoma
ORR: objective response rate
CPT1A: carnitine palmitoyltransferase 1A
KAT2A: lysine acetyltransferase 2A
SIRT5: Sirtuin5
SIRT7: Sirtuin7
GC: gastric cancer
PDAC: pancreatic ductal adenocarcinoma
SOD1: superoxide dismutase
PKM2: pyruvate kinase M2
SHMT2: serine hydroxymethyltransferase2
GLS: glutaminase
SDHA: succinate dehydrogenase complex subunit A
ACOX1: acyl-CoA oxidase 1
TCGA: The Cancer Genome Atlas
CPTAC: Clinical Proteomic Tumor Analysis Consortium
ROC: receiver operating characteristic
IGP: in-group-proportion
PCA: principal component analysis
GO: gene ontology
KEEG: Kyoto Encyclopedia of Genes and Genomes
LASSO: least absolute shrinkage and selection operator
PLMD: Protein Lysine Modifications Database
FBS: fetal bovine serum
NC: negative control
ESCA: esophageal carcinoma
HNSCC: head and neck squamous cell carcinoma
LUSC: lung squamous cell carcinoma
LUAD: lung adenocarcinoma
LIHC: liver hepatocellular carcinoma
KIRP: kidney renal papillary cell carcinoma
KIRC: kidney renal clear cell carcinoma
COAD: colon adenocarcinoma
BRCA: breast invasive carcinoma
BLCA: bladder urothelial carcinoma
AUC: area under curve
CDF: cumulative distribution function
FC: fold change
BP: biological process
CC: cellular component
MF: molecular function
METTL3: methyltransferase like 3
METTL14: methyltransferase like 14
METTL16: methyltransferase like 16
WTAP: Wilms tumor 1 associated protein
VIRMA: Vir like m6A methyltransferase associated
RBM15: RNA binding motif protein 15
RBM15B: RNA binding motif protein 15B
ZC3H13: zinc finger CCCH-type containing 13
FTO: fat mass and obesity associated protein
ALKBH5: AlkB Homolog 5, RNA Demethylase
YTHDF1: YTH N(6)-methyladenosine RNA binding protein 1
YTHDF2: YTH N(6)-methyladenosine RNA binding protein 2
YTHDF3: YTH N(6)-methyladenosine RNA binding protein 3
YTHDC1: YTH domain containing 1
YTHDC2: YTH domain containing 2
AGO2: argonaute RISC catalytic component 2
RBMX: RNA binding motif protein X-linked
ELAVL1: ELAV like RNA binding protein 1
HNRNPA2B1: heterogeneous nuclear ribonucleoprotein A2/B1
HNRNPC: heterogeneous nuclear ribonucleoprotein C (C1/C2)
FMR1: fragile X mental retardation 1
LRPPRC: leucine-rich pentatricopeptide repeat containing
IGF2BP1: insulin-like growth factor 2 mRNA binding protein 1
IGF2BP2: insulin-like growth factor 2 mRNA binding protein 2
IGF2BP3: insulin-like growth factor 2 mRNA binding protein 3
EIF3A: eukaryotic translation initiation factor 3 subunit A
EIF3B: eukaryotic translation initiation factor 3 subunit B
EIF3C: eukaryotic translation initiation factor 3 subunit C
EIF3H: eukaryotic translation initiation factor 3 subunit H
ZCCHC4: zinc finger CCHC domain containing 4
METTL5: methyltransferase like 5
TRMT112: tRNA methyltransferase 11-2 homolog.
