# Supplementary material for: Succinylation Regulators Promote Clear Cell Renal Cell Carcinoma by Immune Regulation and RNA N6-Methyladenosine Methylation
Source: Front Cell Dev Biol. 2021 Feb 18;9:622198. doi: 10.3389/fcell.2021.622198 (PMC7935513; doi:10.3389/fcell.2021.622198)
Supplement: Supplementary file 1 [file Data_Sheet_1.pdf]

Supplementary Material

Supplementary Figures

FIGURE S1 | The flowchart of the whole study. ★ Labelled parts were analyzed by dataset 1 and validated by dataset 2. KIRC, kidney renal clear cell carcinoma; ccRCC, clear cell renal cell carcinoma; TCGA, The Cancer Genome Atlas; DEGs, differently expressed genes; LASSO, least absolute shrinkage and selection operator; LASSO, least absolute shrinkage and selection operator.

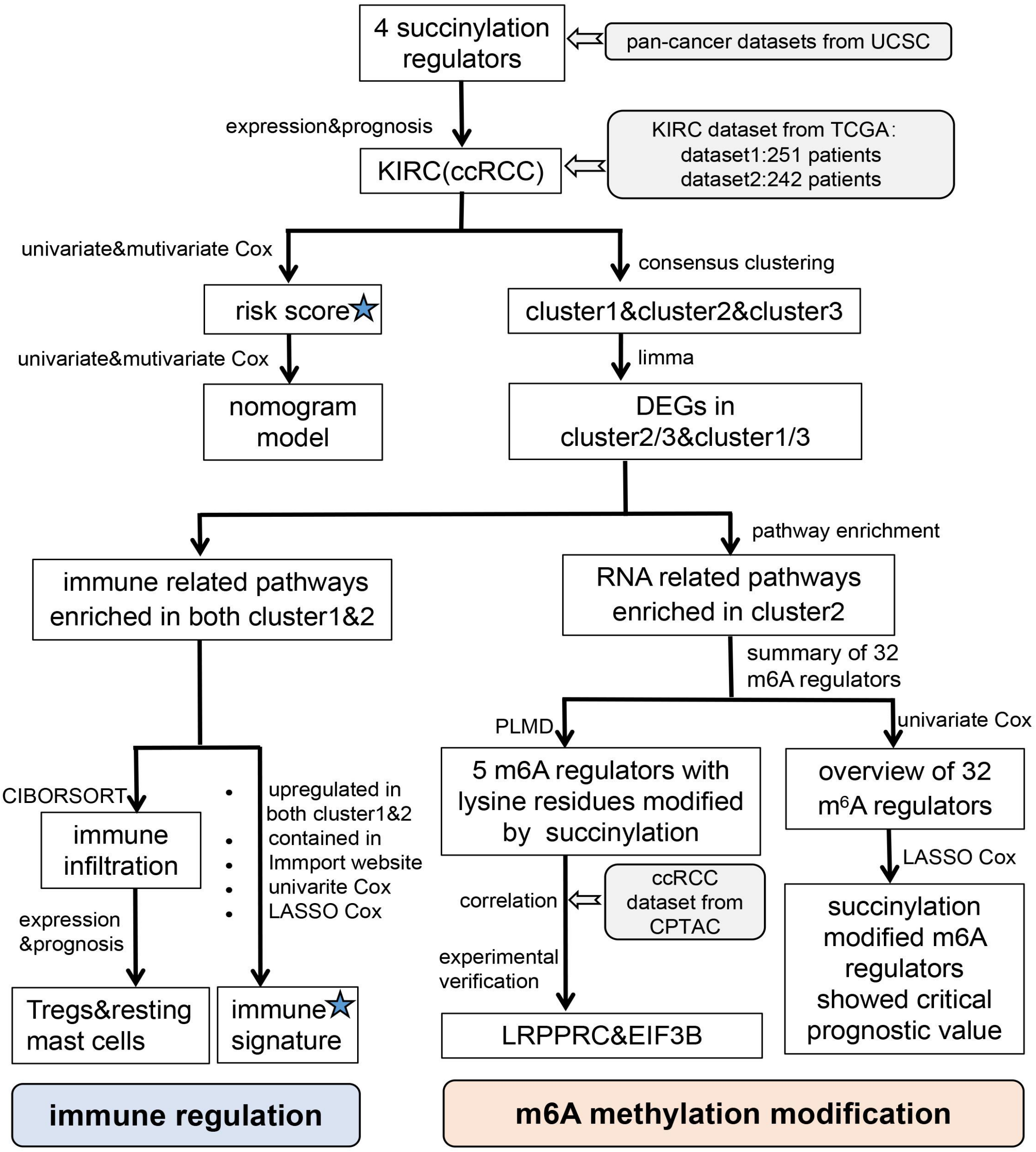

FIGURE S2 | Comparison of clinical characteristics including between dataset 1 and dataset 2. Comparison of Gender **(A)**, Age **(B)**, T stage **(C)**, M stage **(D)**, survival status **(E)** and OS **(F)** between dataset 1 and dataset 2. OS, overall survival.

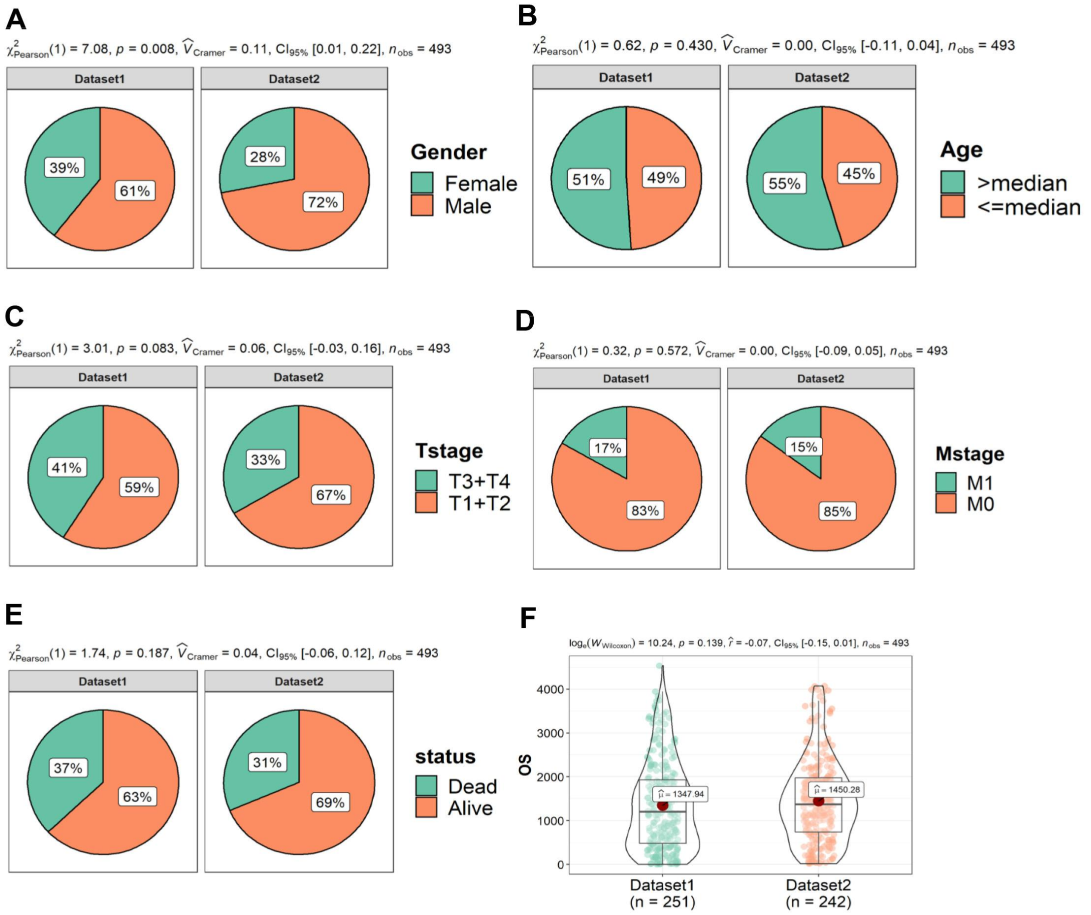

FIGURE S3 | Validation of RS based on succinylation regulators by dataset 2. **(A)** Distribution of the risk score, OS, survival status, and the relative mRNA expression of CPT1A and KAT2A among ccRCC patients in dataset 2. **(B)** Kaplan-Meier survival curves for ccRCC patients with high and low RS in dataset 2. RS, risk score. ccRCC, clear cell renal cell carcinoma.

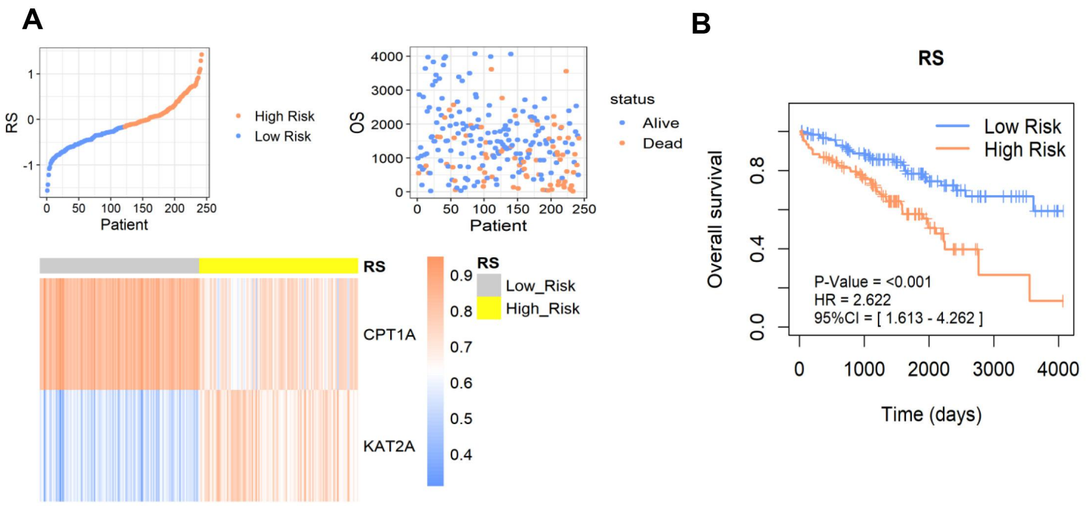

FIGURE S4 | Validation of consensus clustering based on succinylation regulators by dataset 2. **(A)** The IGP values of three clusters in dataset 2 divided by the centroid of clusters in dataset 1. **(B)** The different expression pattern of four succinylation regulators among three clusters in dataset 2, ****$P < 0.0001$. **(C)** PCA of the total mRNA expression profile in dataset 2. **(D)** Kaplan-Meier survival curve for ccRCC patients among different clusters in dataset 2. **(E)** The heatmap demonstrated the expression of four succinylation regulators in patients contained in dataset 2 as well as the association between RS and consensus clusters. PCA, principal component analysis; IGP, in-group-proportion; RS, risk score.

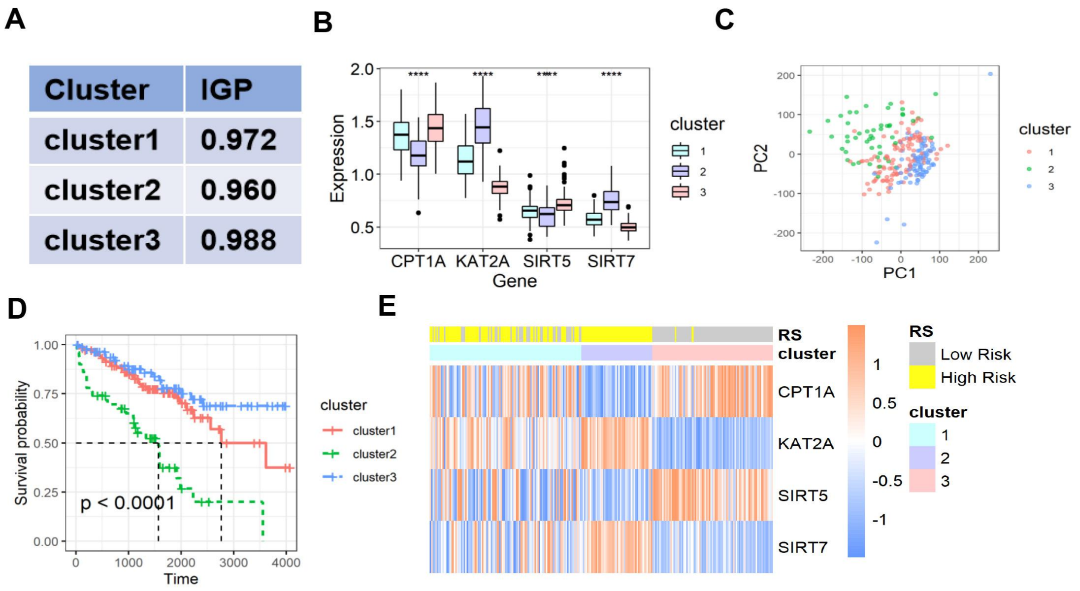

FIGURE S5 | Validation of the immune signature by dataset 2. **(A)** Kaplan-Meier survival curves for ccRCC patients with high and low RS_immune in dataset 2. **(B)** The ROC curves based on immune signature for 1-, 3-, 5- and 8-year survival probabilities analyzed by dataset 2. *P* value for survival curve was calculated by the log-rank test. ccRCC, clear cell renal cell carcinoma; RS, risk score; ROC, receiver operating characteristic.

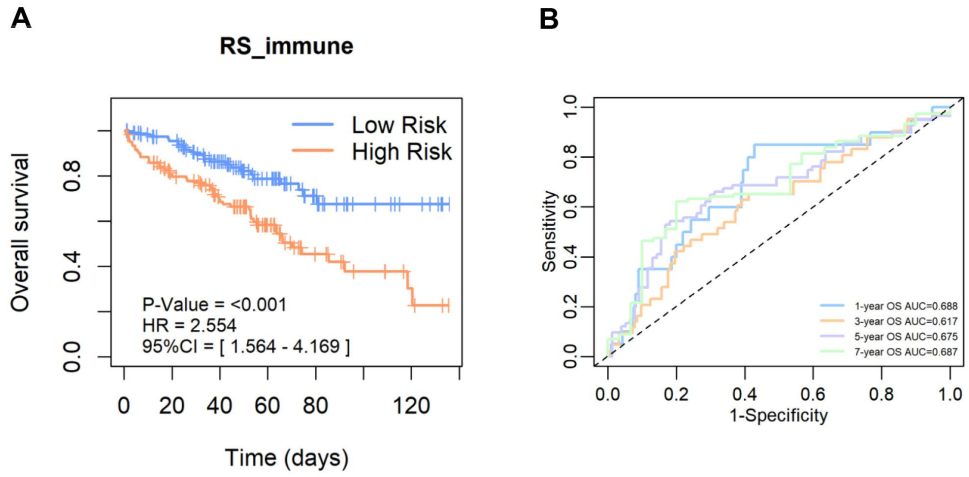

FIGURE S6 | A brief graph summary of the potential mechanism on how succinylation modification promotes malignant progression of ccRCC. In ccRCC, four well-known succinylation regulators work together to regulate the succinylation level of their substrate proteins, among which CPT1A and SIRT5 are downregulated while KAT2A and SIRT7 are upregulated compared to the correspoding normal tissues. Succinylation modification might promote progression of ccRCC in the following manners:①by altering the infiltration of immune cells, such as accelerating the infiltration of Tregs and decreasing the infiltration of M0 macrophages; ② by regulating the expression of m6A regulators including LRPPRC and EIF3B.

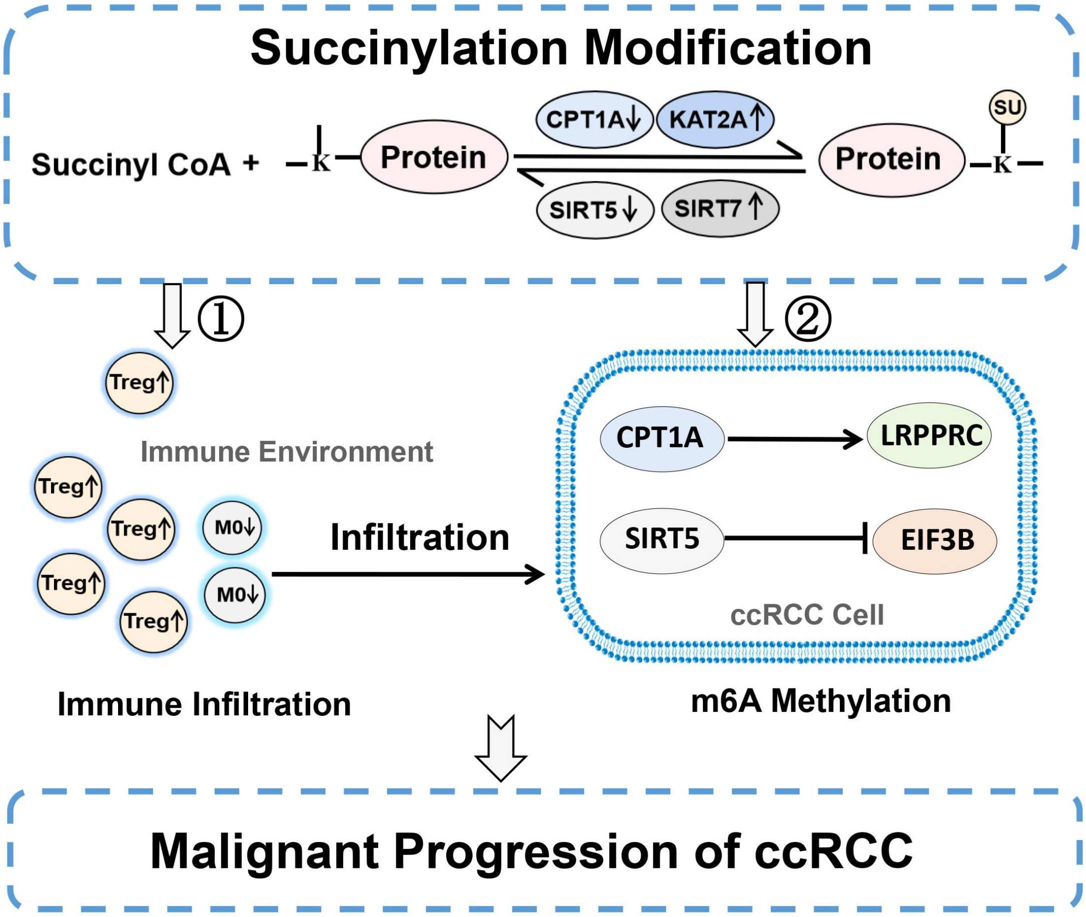
Succinylation Modification
Succinyl CoA +
K
Protein
CPT1A↓
KAT2A↑
SIRT5↓
SIRT7↑
Protein
SU
K
①
②
Treg↑
Immune Environment
Treg↑
Treg↑
M0↓
Treg↑
M0↓
Treg↑
Infiltration
CPT1A
LRPPRC
SIRT5
EIF3B
ccRCC Cell
Immune Infiltration
m6A Methylation
Malignant Progression of ccRCC

Supplementary Tables

TABLE S1 | Clinicopathological characteristics of 251 ccRCC patients in dataset 1.

Parameters	Alive (N=158)	Dead (N=93)	Total (N=251)
Age			
<=median	86 (54.4%)	37 (39.8%)	123 (49.0%)
>median	72 (45.6%)	56 (60.2%)	128 (51.0%)
Gender			
Male	96 (60.8%)	56 (60.2%)	152 (60.6%)
Female	62 (39.2%)	37 (39.8%)	99 (39.4%)
T stage			
T1+T2	111 (70.3%)	37 (39.8%)	148 (59.0%)
T3+T4	47 (29.7%)	56 (60.2%)	103 (41.0%)
N stage			
N0	153 (96.8%)	82 (88.2%)	235 (93.6%)
N1	5 (3.2%)	11 (11.8%)	16 (6.4%)
M stage			
M0	148 (93.7%)	61 (65.6%)	209 (83.3%)
M1	10 (6.3%)	32 (34.4%)	42 (16.7%)

The median age of ccRCC patients in dataset 1 is 62 years old.

TABLE S2 | Clinicopathological characteristics of 242 ccRCC patients in dataset 2.

Parameters	Alive (N=166)	Dead (N=76)	Total (N=242)
Age			
<=median	89 (53.6%)	21 (27.6%)	110 (45.5%)
>median	77 (46.4%)	55 (72.4%)	132 (54.5%)
Gender			
Male	123 (74.1%)	51 (67.1%)	174 (71.9%)
Female	43 (25.9%)	25 (32.9%)	68 (28.1%)
Tstage			
T1+T2	130 (78.3%)	31 (40.8%)	161 (66.5%)
T3+T4	36 (21.7%)	45 (59.2%)	81 (33.5%)
Mstage			
M0	161 (97.0%)	45 (59.2%)	206 (85.1%)
M1	5 (3.0%)	31 (40.8%)	36 (14.9%)

The median age of ccRCC patients in dataset 2 is 60 years old.

TABLE S3 | Clinicopathological characteristics of 42 ccRCC patients for immunohistochemistory.

Parameters	Total(N=42)
Age (years)	

<=median	23 (46.0%)
>median	27 (54.0%)
Gender	
Male	28 (56.0%)
Female	22 (44.0%)
Stage	
I	5 (10.0%)
II	25 (50.0%)
III	15 (30.0%)
IV	5 (10.0%)

The median age of ccRCC patients for immunohistochemistory is 59 years old.

TABLE S4 | The univariate Cox regression analysis between 4 succinylation regulators and OS in 10 common tumors.

Tumor	Gene	HR	95%CI	P
ESCA	CPT1A	1.043	0.673-1.616	0.851
	KAT2A	0.710	0.457-1.103	0.128
	SIRT5	0.872	0.561-1.355	0.541
	SIRT7	0.925	0.593-1.443	0.73
STAD	CPT1A	0.986	0.830-1.172	0.875
	KAT2A	0.909	0.756-1.092	0.309
	SIRT5	0.869	0.668-1.131	0.298
	SIRT7	0.824	0.660-1.029	0.088
LUSC	CPT1A	1.091	0.953-1.249	0.206
	KAT2A	0.888	0.778-1.015	0.081
	SIRT5	0.930	0.778-1.111	0.422
	SIRT7	0.986	0.835-1.166	0.873
LUAD	CPT1A	0.955	0.816-1.118	0.568
	KAT2A	0.972	0.842-1.121	0.692
	SIRT5	1.071	0.840-1.365	0.581
	SIRT7	1.181	0.963-1.448	0.11
LIHC	CPT1A	0.983	0.855-1.131	0.814
	KAT2A	0.989	0.853-1.148	0.887
	SIRT5	0.955	0.781-1.168	0.657
	SIRT7	1.108	0.932-1.318	0.244
KIRP	CPT1A	1.011	0.771-1.327	0.935
	KAT2A	1.118	0.863-1.448	0.399
	SIRT5	0.576	0.365-0.910	0.018
	SIRT7	1.361	0.848-2.184	0.201
KIRC	CPT1A	0.660	0.573-0.759	<0.001
	KAT2A	1.487	1.277-1.732	<0.001
	SIRT5	0.633	0.522-0.766	<0.001
	SIRT7	1.702	1.299-2.229	<0.001

COAD	CPT1A	0.981	0.735-1.309	0.897
	KAT2A	1.009	0.776-1.313	0.947
	SIRT5	1.199	0.770-1.866	0.421
	SIRT7	0.954	0.646-1.408	0.811
BRCA	CPT1A	1.326	1.154-1.524	<0.001
	KAT2A	0.954	0.797-1.141	0.603
	SIRT5	1.170	0.921-1.486	0.198
	SIRT7	0.692	0.571-0.837	<0.001
BLCA	CPT1A	1.148	1.024-1.287	0.018
	KAT2A	0.761	0.617-0.940	0.011
	SIRT5	0.805	0.646-1.004	0.055
	SIRT7	0.743	0.596-0.925	0.008

TABLE S5 | The correlation between the expression of four succinylation regulators and clinicopathological parameters in dataset 1.

Parameter	Total	CPT1A			KAT2A			SIRT5			SIRT7		
		Low	High	P	Low	High	P	Low	High	P	Low	High	P
T stage				<0.001			0.473			0.004			0.024
T1+T2	148	57	91		77	71		62	86		83	65	
T3+T4	103	68	35		48	55		63	40		42	61	
N stage				0.068			0.809			0.429			0.202
N0	235	113	122		118	117		115	120		120	115	
N1	16	12	4		7	9		10	6		5	11	
M stage				0.026			0.135			0.010			0.004
M0	209	97	112		109	100		96	113		113	96	
M1	42	28	14		16	26		29	13		12	30	
Age				0.951			0.951			0.487			0.658
<median	123	62	61		62	61		58	65		59	64	
>=median	128	63	65		63	65		67	61		66	62	
Gender				<0.001			0.326			0.959			0.278
female	99	35	64		45	54		50	49		54	45	
male	152	90	62		80	72		75	77		71	81	

The median age of ccRCC patients in dataset 1 is 62 years old.
